# Perceived clinical and ethical impact of digital transformation in healthcare and research: A survey in the MENA region

**DOI:** 10.1371/journal.pone.0336618

**Published:** 2025-12-02

**Authors:** Hisham E. Hasan, Karem H. Alzoubi, Omar F. Khabour, Wael K. Al-Delaimy, Kenneth W. Goodman

**Affiliations:** 1 Department of Clinical Pharmacy, Faculty of Pharmacy, Jordan University of Science and Technology, Irbid, Jordan; 2 Department of Pharmaceutical Sciences, College of Pharmacy, QU Health, Qatar University, Doha, Qatar; 3 Faculty of Pharmacy, Jordan University of Science and Technology, Irbid, Jordan; 4 Department of Medical Laboratory Sciences, Faculty of Applied Medical Sciences, Jordan University of Science and Technology, Irbid, Jordan; 5 Herbert Wertheim School of Public Health and Human Longevity, UC San Diego Division of Global Health, San Diego, United States of America; 6 Institute for Bioethics and Health Policy, University of Miami Miller School of Medicine, Miami, United States of America; Dana-Farber Cancer Institute, Harvard Medical School and THE Broad Institute of MIT and Harvard, LEBANON

## Abstract

**Background:**

The shift toward digital medical records and communication represents a transformative change in healthcare practices and research. However, this transition is marked by challenges related to the ethics of digital health adoption in fragile and conflict-affected areas, particularly in areas where healthcare providers lack prior training and preparation.

**Objective:**

To assess healthcare and research professionals’ adoption, attitudes, perceptions, and ethical considerations toward digital transformation in healthcare and research across the Middle East and North Africa (MENA) region.

**Methods:**

A cross-sectional survey was conducted in 20 MENA countries involving 990 health professionals and researchers. Demographic data was collected, as well as questions related to the impact, attitudes, and ethics of digital health transformation. Data were analyzed using descriptive and inferential statistical methods to identify patterns and correlations between sociodemographic factors and responses.

**Results:**

The results indicate that digital health transformation in the MENA region was perceived as limited to moderate across key domains, with relatively positive scores on attitudes and ethical considerations. While foundational technologies, such as electronic health records, show moderate adoption, other advanced tools remain underutilized. The study also revealed a significant lack of formal training in digital health (*p* < 0.001) despite high digital proficiency among participants (*p* < 0.001). However, ethical concerns were highlighted as key issues with strong support for enhanced bioethics literacy. These findings align with the Sustainable Development Goals (SDGs) and emphasize the need to develop inclusive policies, implement robust regulatory frameworks, create targeted educational programs, make infrastructure investments, and foster stakeholder collaboration to promote the ethical use of digital health technologies.

**Conclusion:**

Digital transformation in healthcare and research is viewed positively across the MENA region, but significant gaps remain in adoption, training, and governance. Addressing these gaps will be essential to ensure safe, equitable, and sustainable digital health implementation.

## Introduction

The rapid advancement of digital health technologies has transformed healthcare. Optimists believe this change will lead to an era of personalized, participative, preventive, predictive, and precision medicine (also known as “5P medicine”). This shift promises to revolutionize healthcare delivery by tailoring treatments to individual characteristics and needs [1]. In high-income countries, digital transformation has become a cornerstone of modern healthcare by offering new opportunities to enhance research practices, health outcomes, and solve longstanding problems [2,3]. This intersection between technology and personalized medicine aims to customize healthcare based on individual or group characteristics, utilizing technologies like mobile health, the internet of things, virtual reality (VR), and artificial intelligence (AI) [4,5].

In the Middle East and North Africa (MENA) region, the adoption of digital health technologies has been gradually increasing, driven by a growing recognition of their potential to improve healthcare delivery [6,7]. Leading this effort is the MENA Health Informatics Association (MENAHIA), which advocates for inclusive digital health initiatives. It emphasizes the importance of digital inclusion, ensuring accessibility to technology, infrastructure, skills, and usability [8]. As the region struggles with challenges such as the rising burden of chronic diseases, inadequate healthcare infrastructure, workforce shortages, socioeconomic diversity, and disparities in access to healthcare services, digital health initiatives can address these challenges by improving healthcare accessibility, efficiency, and quality [9].

The COVID-19 pandemic highlighted the urgent need to utilize technology to sustain and strengthen healthcare systems, acting as a catalyst for digital health adoption [10]. From electronic health records (EHRs) to telemedicine platforms, countries such as Egypt, Jordan, and those in the Gulf Cooperation Council have been increasingly integrating digital solutions to bridge gaps in healthcare delivery and improve access to services [8]. Rapid digitalization also poses many ethical challenges that require careful consideration and proactive measures to safeguard the rights and well-being of patients and participants involved in research studies [11–13].

Medical practice, public health, and health research all raise ethical issues [1,14]. Consequentialism, deontology, virtue ethics, and religious inspiration have been used to guide decision-making. All these approaches, systems, and theories need to be balanced against technical and contextual considerations [1,15]. Scholars increasingly argue that digital transformation represents not merely incremental digitalization but a paradigm shift in healthcare systems—disruptive, radical, and holistic in scope [16,17]. While digital health technology adoption promises opportunities, it is also widely agreed to pose challenges related to privacy, data security, and equitable access to care [18]. An emerging ethical debate concerns the tension between utilitarian approaches, which emphasize overall benefit, and prioritarian approaches, which prioritize the needs of the most disadvantaged—an especially relevant distinction in the MENA region, where refugees, displaced persons, and populations in conflict-affected settings remain at risk of exclusion [19,20].

Personalized digital health operates within a complex ecosystem influenced by socio-cultural, structural, international, and situational factors. Understanding context is crucial for effective implementation, considering factors such as societal attitudes, policy readiness, economic conditions, and global connectivity [1,15,21]. Ethical decisions in digital health systems must be evidence-based, consider innovative potential, and mitigate risks. Monitoring and evaluation are essential for assessing system performance and identifying areas for improvement, while fostering a culture of no-blame reporting ensures continuous refinement and trust in the system [1,22].

However, stakeholders’ views on how this transformation affects clinical practice and ethical considerations remain underexplored. This study investigates the opportunities and challenges of digital transformation across the MENA region. Specifically, we conducted a cross-sectional survey to evaluate attitudes, perceptions, and ethical concerns regarding digital health adoption among healthcare and research professionals in 20 countries. Our aim is to inform policymakers, healthcare providers, researchers, and other stakeholders on how to navigate digital transformation while upholding ethical principles and ensuring equitable healthcare delivery. To this end, we examine the current level of digital health adoption, assess stakeholders’ perspectives on its impact on health outcomes and well-being, explore ethical concerns, and provide practical recommendations.

## Materials and methods

### Study design

This cross-sectional research employed quantitative methods to investigate digital transformation in healthcare within the MENA region. A thorough review of relevant literature with reference to the World Health Organization’s six ethical principles guidance informed the development of a survey aimed at assessing perspectives on digital transformation and ethical considerations among stakeholders [1,2,5,15,23–25]. The online questionnaire/survey was active from June 1, 2024, to September 17, 2024, and was designed for completion within 10–15 minutes (S1 Appendix). To enhance survey reliability and validity, strategies included randomizing response options and considering biases such as social desirability or acquiescence bias and order effects in multiple-choice questions. This study was conducted and reported in accordance with the STROBE guidelines for cross-sectional studies.

### Sampling strategy

The study utilized purposive sampling to target key stakeholders—researchers, healthcare providers, policymakers, and technology developers—from diverse backgrounds across the MENA region. We were able to reach a sample size of 900, exceeding the minimum requirement of 785 calculated using Raosoft’s sample size calculator at a 95% confidence level and 5% margin of error [26]. The inclusion criteria included being aged 18 or older, associated with a MENA country, involved in healthcare or research, and being a native Arabic speaker. Incomplete responses were excluded.

### Study instrument and data collection

The questionnaire was uploaded to Google Forms as an anonymous online survey, available in both English and Arabic. The survey consisted of multiple-choice questions and a 5-point Likert scale (strongly agree, agree, neutral, disagree, strongly disagree) covering several domains, including socio-demographic information, attitudes towards the adoption of digital health, perceptions of digital health impact on research practices, and ethics. Content and face validity were ensured through expert review (6 PhD experts in clinical research practices, computer information systems in Arabic, digital health, and bioethics), aligning each question with the research objectives and accommodating the diverse background of respondents. Before widespread distribution, a pilot test with a small sample (*n* = 15) ensured clarity and technical functionality of the survey. Distribution methods included dissemination through academic institutions, research centers, and hospitals across the region. Most participants were recruited from academic profiles published on various institutional websites, including universities and teaching hospitals, to ensure a broad scope. Participants were invited via email or social media platforms, using either a QR code or a direct link to the survey website.

### Ethical approval

This research was approved by the Jordan University of Science and Technology (JUST) and King Abdullah University Hospital (KAUH) IRB prior to data collection (approval reference number: 62/170/2024) on May 28^th^, 2024. The approved protocol included the questionnaire and study methodology. All procedures were conducted in accordance with the Declaration of Helsinki and its later amendments. Electronically written informed consent was obtained from all participants, and measures were taken to ensure that respondents provided honest answers by emphasizing the anonymity and confidentiality of their responses. Access to the collected data was restricted to the research team members in compliance with KAUH IRB regulations (policy number: GM7601).

### Data analysis

**Fig 1:**
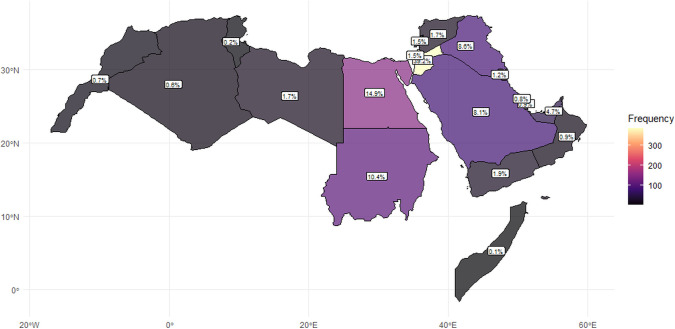
Geographic distribution of respondents across the MENA region. Map showing the percentage of participants from each country, illustrating the relative contribution of each national sample to the overall study population. Base map data source: Natural Earth (public domain; https://www.naturalearthdata.com). Generated in R (v4.4) using the *rnaturalearth* and *sf* packages.

Data were managed and analyzed using IBM SPSS software (version 27), reporting frequencies (*n*), percentages (%), means (*m*), and standard deviations (SD) as appropriate. All items were fully completed by participants; no missing data were present. The responses from participants were used to calculate normalized scores for each of the major sections of the survey. The overall score for digital health adoption and transformation in the MENA region was calculated based on participants’ responses to survey items measuring their experiences and perceptions regarding digital health technologies. Attitudes and ethical considerations scores were similarly calculated based on participants’ ratings of relevant statements. The normalized scores were computed by dividing the sum of the responses for each category by the maximum possible score, scaling them to a range from 0 to 1. Inferential statistics and regression analyses were conducted for subgroup analysis. A *p-*value below 0.05 was considered statistically significant. Figures were designed using R software (version 4.4) with the *tidyverse*, *rnaturalearth*, and *sf* packages. The underlying shapefiles of the regional map (Fig 1) are from Natural Earth, which are in the public domain (CC0 license). In the pilot study group, the Cronbach’s alpha coefficients assessed internal consistency across Likert scale items (5, 10, and 15), confirming strong reliability (α = 0.815, 0.927, and 0.950, respectively) for the three sets of variables.

## Results

### Sociodemographic profile

Participants in the study were asked to identify the country in which they lived or worked during the study period. From a total of 990 valid responses, the most frequently mentioned country was Jordan (39.2%), followed by Egypt (14.9%) and Sudan (10.4%). Participants from other countries in the region were Iraq (8.6%), Saudi Arabia (8.1%), and the United Arab Emirates (4.7%). Smaller counts were noted for additional countries listed in S1 Table of S2 Appendix. The geographical distribution of these participants is illustrated in Fig 1.

Table 1 describes the participants’ sociodemographic characteristics. Healthcare providers comprised the majority (68.4%), followed by biomedical researchers or academics (22.8%), and other health-related professionals (8.8%). Among healthcare providers, physicians were the most common (40.8%), followed by pharmacists (28.6%) and allied health professionals (20.4%), with nurses making up 10.2%. A significant portion (81.3%) lived in urban areas and consisted of females (65.3%). The average age was 31.1 years; most reported middle-income levels (60.9%). Educationally, 61.4% held bachelor’s degrees, and 78.8% reported not having received adequate training in digital health topics. Furthermore, 58.3% rated their proficiency in digital technologies as high or very high, and the majority had research experience for 1 year or more (40.8%).

**Table 1:** Sociodemographic characteristics of study participants (n = 990).

Item		Total *n* = 990	Profession,* n* (%)			*p-*value^a^
			Healthcare providers *n* = 677 (68.4)	Medical researchers/ Academics *n* = 226 (22.8)	Others *n* = 87 (8.8)	
Geographic location	Urban	805 (81.3)	553 (81.7)	186 (82.3)	66 (75.9)	0.385
	Rural	185 (18.7)	124 (18.3)	40 (17.7)	21 (24.1)	
Gender	Male	344 (34.7)	197 (29.1)	106 (46.9)	41 (47.1)	<0.001
	Female	646 (65.3)	480 (70.9)	120 (53.1)	46 (52.9)	
Age (Years)	*m* ± SD	31.1 ± 10.6	28.8 ± 9.3	38.4 ± 11.4	29.5 ± 9.5	<0.001
Income level (depending on the country’s wage system)^b^	Lower income	302 (30.5)	226 (33.4)	49 (21.7)	27 (31.0)	0.003
	Middle income	603 (60.9)	401 (59.2)	147 (65.0)	55 (63.2)	
	Upper income	85 (8.6)	50 (7.4)	30 (13.3)	5 (5.8)	
Educational background (highest completed level of education)	Bachelor’s degree	608 (61.4)	506 (74.7)	41 (18.1)	61 (70.1)	<0.001
	Master’s degree	198 (20.0)	110 (16.3)	71 (31.4)	17 (19.6)	
	Doctor of Philosophy (PhD)	184 (18.6)	61 (9.0)	114 (50.5)	9 (10.3)	
Employment setting (work environment)	Unemployed	104 (10.5)	79 (11.7)	14 (6.2)	11 (12.7)	<0.001
	Internship/trainee	328 (33.1)	266 (39.3)	29 (12.8)	33 (37.9)	
	Hospital or clinic	152 (15.4)	142 (21.0)	2 (0.9)	8 (9.2)	
	Private business (pharmacy, laboratory, etc.)	83 (8.4)	71 (10.5)	10 (4.4)	2 (2.3)	
	Academic or research institution	223 (22.5)	66 (9.7)	144 (63.7)	13 (14.9)	
	Entrepreneurial company or industry	29 (2.9)	19 (2.8)	2 (0.9)	8 (9.2)	
	Government agency (Ministry of Health, FDA, etc.)	53 (5.4)	25 (3.7)	20 (8.9)	8 (9.2)	
	Non-governmental organization (NGO)	18 (1.8)	9 (1.3)	5 (2.2)	4 (4.6)	
Professional sector affiliation	Not applicable	194 (19.6)	170 (25.1)	4 (1.7)	20 (23.0)	<0.001
	Public (governmental)	430 (43.4)	266 (39.3)	126 (55.8)	38 (43.7)	
	Private	366 (37.0)	241 (35.6)	96 (42.5)	29 (33.3)	
English proficiency^b^	Fluent	248 (25.1)	158 (23.3)	72 (31.9)	18 (20.7)	0.012
	Proficient	503 (50.8)	353 (52.2)	111 (49.1)	39 (44.8)	
	Basic	239 (24.1)	166 (24.5)	43 (19.0)	30 (34.5)	
Digital technologies utilization proficiency^b^	Very high	139 (14.0)	76 (11.2)	42 (18.6)	21 (24.1)	0.001
	High	438 (44.3)	293 (43.3)	110 (48.7)	35 (40.2)	
	Neutral	316 (31.9)	235 (34.7)	59 (26.1)	22 (25.3)	
	Low	84 (8.5)	62 (9.2)	15 (6.6)	7 (8.1)	
	Very low	13 (1.3)	11 (1.6)	0 (0.0)	2 (2.3)	
Received any formal training or education on digital health topics	Yes	210 (21.2)	132 (19.5)	50 (22.1)	28 (32.2)	0.023
	No	780 (78.8)	545 (80.5)	176 (77.9)	59 (67.8)	
Years of experience in research	None/ Not applicable	331 (33.4)	283 (41.8)	12 (5.3)	36 (41.4)	<0.001
	Less than 1 year	255 (25.8)	200 (29.5)	30 (13.3)	25 (28.7)	
	1-5 years	200 (20.2)	123 (18.2)	61 (27.0)	16 (18.4)	
	6-10 years	69 (7.0)	24 (3.6)	39 (17.2)	6 (6.9)	
	More than 10 years	135 (13.6)	47 (6.9)	84 (37.2)	4 (4.6)	

^a^A *p-*value of less than 0.05 indicates statistical significance, calculated by the *chi*-square test.

^b^Participants self-rated.

### Digital health transformation

**Fig 2:**
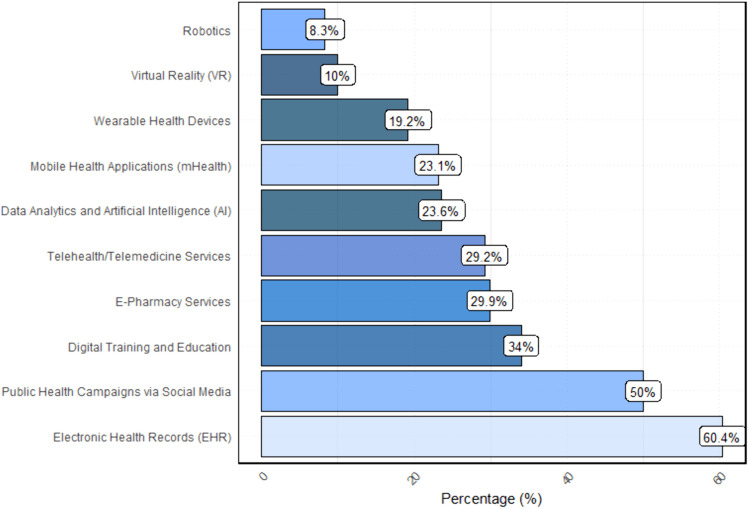
Distribution of prevalent digital health technologies used in healthcare/research reported by participants across MENA countries. Bar chart illustrates the percentage of respondents reporting adoption of various digital health tools. Multiple responses were allowed. EHRs dominated adoption, while VR and robotics showed the lowest levels of uptake, reflecting uneven maturity of different technologies in the region.

Fig 2 shows categories of commonly used digital health technologies in participants’ countries. The most frequently selected category was EHRs, with 598 responses, representing 60.4%. This was followed by public health campaigns through social media (50.0%) and digital training and education (34.0%). E-pharmacy services and telehealth services were chosen by 29.9% and 29.2%, respectively. Data analytics and AI garnered 23.6%, mobile health (mHealth) applications received 23.1%, and wearable health devices attracted 19.2%; while VR and robotics had the lowest adoption, with only 99 (10.0%) and 82 (8.3%) selections, respectively.

S2 Table in S2 Appendix assesses participants’ familiarity and satisfaction levels with the current digital transformation in healthcare. Regarding familiarity, 39.1% reported being familiar with the concept of digital transformation and 32.4% indicated they were not. Similarly, familiarity with its presence in research settings was noted by 36.0%, while many remained neutral toward digital health terminologies (34.6%). However, a majority expressed being dissatisfied with the current level of digital health adoption in their institutions (46.8%) and the incorporation of emerging technologies (45.1%).

S3 Table in S2 Appendix reports participants’ awareness and involvement in initiatives driving digital transformation in healthcare within the MENA region. Only 29.5% reported being aware of major governmental or large-scale regional programs aimed at this transformation, while a majority were not (70.5%). Regarding regional infrastructure readiness, 54.3% believed that the necessary support for digital transformation was lacking. Participation in clinical research utilizing digital technology was low, with only 17.9% affirming their involvement. However, 43.8% said they believed that sufficient research evidence supports the adoption of digital health technologies. In terms of personal engagement, 61.6% reported downloading and using health or wellness mobile apps, but awareness of regulatory guidelines for digital tools in clinical trials was limited, with only one-third expressing familiarity (33.0%).

### Attitudes towards digital health in research

**Fig 3:**
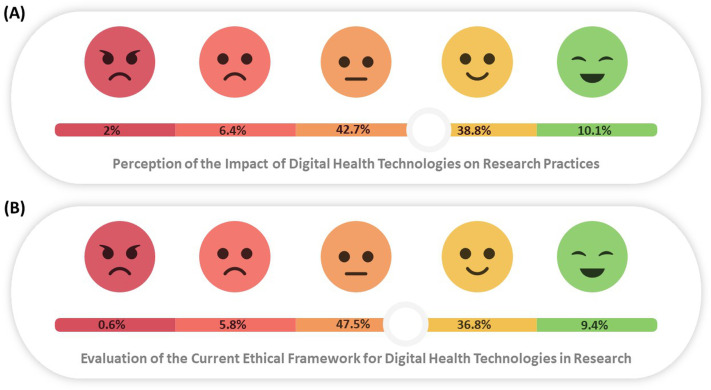
Perspectives on digital health technologies. Two panels show participant ratings on a 5-point Likert scale (ranging from very negative to very positive). **(A)** Overall perception of the advantages and barriers (mean 3.5 ± 0.8). **(B)** Perception of ethical framework application and suggested solutions (mean 3.5 ± 0.8). Percentages of responses are displayed, highlighting both supportive and critical views.

Fig 3 illustrates the spectrum of perceptions among participants regarding the impact of digital health technologies on research practices in their region. Most respondents expressed a neutral stance, with 423 selections (42.7%). A combined positive evaluation response accounted for 48.9%. Conversely, 8.4% gave a negative assessment. In a related question assessing the application of the current ethical framework for digital health technologies in human research, 47.5% remained neutral. A positive evaluation of the ethical framework was reported by 46.2%, while 6.4% gave a negative evaluation.

Table 2 presents participants’ attitudes towards digital health technologies, highlighting their beliefs about the potential clinical impact on health. A substantial majority agreed that digital technologies could contribute to better health and well-being in the future (68.8%). Additionally, 64.4% believed that these technologies could enhance clinical decision-making within their specialties. Optimism about improving public health outcomes was shared by 65.1%. However, opinions varied regarding the adequacy of training for digital health use, with only 20.8% feeling well-prepared for their roles. Concerns were also raised about algorithm quality, as 53.4% recognized its importance for the effective implementation of digital health technologies. Participants acknowledged potential challenges associated with digital solutions, including cultural resistance, with 60.2% anticipating difficulties in implementation.

**Table 2:** Attitudes towards digital health technologies (n = 990).

Statement	*n* (%)				
	Strongly disagree	Disagree	Neutral	Agree	Strongly agree
I believe that digital technologies have the potential to contribute to better health and well-being in the future	24 (2.4)	57 (5.8)	227 (22.9)	535 (54.0)	147 (14.8)
The use of digital health in my specialty could improve clinical decision-making	21 (2.1)	69 (7.0)	262 (26.5)	504 (50.9)	134 (13.5)
I am optimistic about the use of digital health technologies in improving public health outcomes and healthcare delivery	17 (1.7)	88 (8.9)	240 (24.2)	514 (51.9)	131 (13.2)
The introduction of digital health technologies will reduce the financial costs associated with my role	28 (2.8)	107 (10.8)	297 (30.0)	445 (44.9)	113 (11.4)
Digital health technologies may take over part of my role as a healthcare professional in the future	40 (4.0)	140 (14.1)	289 (29.2)	417 (42.1)	104 (10.5)
I have been adequately trained to use digital health that is specific to my role	175 (17.7)	312 (31.5)	297 (30.0)	169 (17.1)	37 (3.7)
The effectiveness of digital health technologies heavily relies on the quality of their underlying algorithms	33 (3.3)	75 (7.6)	353 (35.7)	425 (42.9)	104 (10.5)
Digital health interventions could effectively address the higher prevalence of the stigma surrounding mental health issues in our communities	31 (3.1)	113 (11.4)	411 (41.5)	363 (36.7)	72 (7.3)
Digital solutions enhance efficiency and effectiveness in clinical research, particularly increasing participation from minorities in remote communities	26 (2.6)	93 (9.4)	300 (30.3)	470 (47.5)	101 (10.2)
I anticipate common challenges when using digital solutions in healthcare and clinical research, such as cultural resistance to change or digitization	36 (3.6)	80 (8.1)	278 (28.1)	474 (47.9)	122 (12.3)

### Ethical considerations in digital transformation

Table 3 outlines participants’ perceptions of various ethical considerations in digital health technologies, revealing a strong consensus on the integration of ethical principles in healthcare system development, with two-thirds advocating for a comprehensive ethical decision-making process (66.7%). Concerns about informed consent in digital research to address urgent public health issues were also notable, with 35.6% agreeing on the importance of having proper consent processes. Regarding privacy and security, 69.0% represented a strong consensus on the crucial roles of both in clinical research. Additionally, 62.6% supported equitable access to digital healthcare as a basic right. Furthermore, 43.5% expressed worries about exacerbating health inequalities, particularly during pandemics. There was also robust support for enhancing bioethics literacy, with 65.9% believing that stakeholders should have a better understanding of bioethics concepts and issues. While 39.0% remained neutral regarding ethical challenges associated with digital health technologies, almost a quarter reported being unaware of these issues (23.0%). Nonetheless, 65.3% acknowledged the need for greater awareness of the social and ethical implications of technological advancements in health research. Overall, these findings reflect a strong awareness of the ethical and societal implications of digital health technologies and the need for policies, ongoing monitoring, and a blame-free reporting culture to foster trust and effective implementation in healthcare settings.

**Table 3:** Ethical considerations in digital health technologies (n = 990).

Statement	*n* (%)				
	Strongly disagree	Disagree	Neutral	Agree	Strongly agree
I believe that digital health technologies should prioritize principles such as respect for autonomy, beneficence, non-maleficence, and justice to provide a comprehensive ethical decision-making process	22 (2.2)	70 (7.1)	238 (24.0)	494 (49.9)	166 (16.8)
I am concerned about ensuring proper informed consent procedures in welfare research conducted using digital tools	37 (3.7)	142 (14.3)	459 (46.4)	292 (29.5)	60 (6.1)
I think that ensuring privacy, confidentiality, security, and fairness in the use of digital health technologies is crucial, especially in clinical research	21 (2.1)	69 (7.0)	217 (21.9)	486 (49.1)	197 (19.9)
I am concerned that even the effective use of medical and personal data in digital healthcare will raise ethical concerns, particularly regarding the protection of the rights and identity of research subjects	35 (3.5)	157 (15.9)	351 (35.5)	353 (35.7)	94 (9.5)
I believe that access to digital health care should be considered a basic right, and efforts must be made to ensure equitable access to necessary infrastructure	26 (2.6)	64 (6.5)	280 (28.3)	488 (49.3)	132 (13.3)
I am concerned about the potential exacerbation of health inequities by digital technologies, especially in the context of pandemics	31 (3.1)	129 (13.0)	400 (40.4)	353 (35.7)	77 (7.8)
I believe that stakeholders in the digital health ecosystem should have a better understanding of bioethical concepts and their application in practice	23 (2.3)	66 (6.7)	249 (25.2)	484 (48.9)	168 (17.0)
I am aware of the ethical issues, challenges, and harms associated with digital health technologies	69 (7.0)	158 (16.0)	386 (39.0)	313 (31.6)	64 (6.5)
Governments should actively resist undue influence from large technology corporations in shaping health-related policies, ensuring public interests are prioritized	27 (2.7)	66 (6.7)	317 (32.0)	425 (42.9)	155 (15.7)
There is a need for greater awareness and consideration of the social and ethical implications of technological advancements in health research	26 (2.6)	62 (6.3)	256 (25.9)	479 (48.4)	167 (16.9)
The quality and functionality of digital healthcare systems depend only on technical aspects and not on their effective adoption and ethical values	74 (7.5)	204 (20.6)	358 (36.2)	292 (29.5)	62 (6.3)
Personalized medicine should be accessible to all individuals based on evidence that reflects their lived experience and biology, treating them equally according to their needs	24 (2.4)	83 (8.4)	295 (29.8)	460 (46.5)	128 (12.9)
I am aware of various approaches, including Islamic religious (*maqāṣid al-sharīʿa*), socio-cultural, and political factors, involved in the transparent and interactive assessment of health technology and innovation	66 (6.7)	136 (13.7)	377 (38.1)	345 (34.8)	66 (6.7)
Regular monitoring of digital health systems is essential to identify problems and ensure adherence to specifications	18 (1.8)	69 (7.0)	266 (26.9)	485 (49.0)	152 (15.4)
Establishing a no-blame reporting culture is crucial for identifying and rectifying problems in digital health systems, ensuring optimal trust and utilization by users	19 (1.9)	63 (6.4)	273 (27.6)	474 (47.9)	161 (16.3)

**Fig 4:**
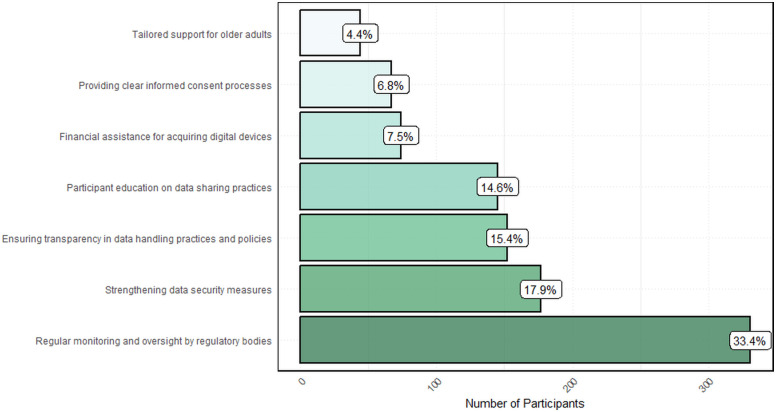
Key measures for the ethical use of digital tools in clinical research (n = 990). Bar chart illustrates the proportion of participants who identified ethical priorities for digital health adoption. While informed consent—historically a major concern—was still noted, participants may view it as relatively well established. In contrast, the strongest emphasis was placed on the need for regular monitoring and oversight by regulatory bodies, highlighting a shift toward systemic governance as the most urgent and practical requirement still lacking in many settings.

Fig 4 illustrates participants’ perspectives on measures needed to promote the ethical utilization of digital tools in clinical research. The most frequently identified action was regular monitoring and oversight by regulatory bodies, representing one-thirds of the total (33.4%). This was followed by strengthening data security measures (17.9%) and ensuring transparency in data handling practices and policies (15.4%). Participant education on data sharing practices was identified by 14.6% of respondents as a necessary measure, while financial assistance for acquiring digital devices (7.5%), providing clear informed consent processes (6.8%), and tailored support for older adults received the least attention (4.4%).

### Comparison of subgroups

S4 Table in S2 Appendix summarizes the normalized scores across key categories in this study, reflecting participants’ perceptions regarding digital health. The overall score for digital health adoption and transformation in the MENA region was 0.41 ± 0.16, indicating some degree of acceptance with limited integration of digital health technologies within practicing healthcare systems. However, attitudes toward digital health transformation scored higher at 0.69 ± 0.13, while ethical considerations received a score of 0.70 ± 0.12, reflecting more positive responses from participants in these areas (strong agreement on both the potential benefits of these technologies and the importance of ethical principles in digital health practices). The *p*-values from the subgroup analyses, including all significant differences, are detailed in S5-S8 Tables in S2 Appendix documents comprehensive data on each subgroup analysis.

S5 Table in S2 Appendix outlines the parameters influencing the calculated scores in the areas of digital health transformation—adoption, attitudes, and ethical considerations. Notably, geographic location significantly affected adoption scores, with urban participants scoring higher than rural counterparts (*p* = 0.004). Although factors such as gender, profession, and income level did not significantly influence adoption, attitudes or ethics scores, lower income was linked to a substantial decrease in adoption scores (*p* < 0.001). Educational background had a marked impact, with participants holding a PhD achieving higher scores in both attitudes and ethical considerations (*p* < 0.05). Additionally, proficiency in English and digital technologies was positively correlated with higher scores across all categories, especially among those with very high digital proficiency (*p* < 0.001). Formal training on digital health topics also positively influenced adoption scores (*p* < 0.001). Participants with over 10 years of research experience exhibited significantly higher attitude and ethical considerations scores (*p* < 0.05).

The multiple linear regression analysis summarized in S6 Table in S2 Appendix identified key predictors of attitudes and ethical considerations regarding digital health in human research. The model exhibited a strong fit across all variables, with significant results (*p* < 0.001) underscoring the relevance of the digital health adoption and transformation score as a predictor (*r* = 0.316, *β* = 0.151, *p* < 0.001). Ethical considerations were significantly affected by both the digital health adoption score (*r* = 0.231, *β* = −0.007, *p* < 0.001) and attitudes towards digital health (*r* = 0.752, *β* = 0.754, *p* < 0.001), highlighting a strong association between ethical perceptions and overall attitudes (*β* = 0.748, *p* < 0.001). These findings emphasize the interrelated nature of attitudes, ethical considerations, and digital health adoption, with positive attitudes playing a crucial role in enhancing ethical perceptions.

Logistic regression analyses in S7 and S8 Tables in S2 Appendix further identified significant predictors of positive attitudes toward digital health transformation and ethical issues. The model was statistically significant (*p* < 0.001). Notably, receiving formal training on digital health topics was associated with lower odds of positive attitudes (OR = 0.547, 95% CI: 0.378–0.792, *p* = 0.001) and reduced moral distress or more ethical concerns (OR = 0.425, 95% CI: 0.295–0.612, *p* < 0.001). Gender also influenced outcomes, with male participants demonstrating fewer ethical precautions compared to females (OR = 0.666, *p* = 0.009). Moreover, the digital health adoption and transformation score emerged as a strong predictor, significantly increasing the odds of positive attitudes (OR = 75.1, 95% CI: 26.8–210.5, *p* < 0.001) and increasing ethical precautions (OR = 19.5, 95% CI: 7.2–52.4, *p* < 0.001). Other variables, including age, job type, income level, educational background, employment type, sector affiliation, English proficiency, and digital proficiency did not show significant predictive value (*p* > 0.05).

## Discussion

The digital transformation in health research presents both significant opportunities for enhancing clinical outcomes and complex ethical challenges that must be addressed. Aligned with the United Nations’ Sustainable Development Goals (SDGs), technological advancements can improve healthcare delivery, expand access, and enhance public health outcomes [27]. However, these potential advancements also entail ethical issues that need careful consideration, especially when applied in diverse and resource-limited settings [28,29].

This study offers a comprehensive exploration of the digital health landscape across the MENA region, examining adoption trends, professional attitudes, and ethical considerations among a diverse sample of healthcare providers and researchers. The findings highlight both the growing recognition of digital health’s potential and persistent gaps in infrastructure, training, and ethical oversight that hinder its effective integration into clinical and research settings.

In this cross-sectional survey of 990 healthcare and research professionals from 20 MENA countries, we found generally positive attitudes toward digital health (mean score 0.69 ± 0.13) and strong recognition of ethical considerations (0.70 ± 0.12), despite relatively modest levels of adoption and transformation (0.41 ± 0.16). Participants who had received formal training reported more cautious attitudes and greater ethical concerns, underscoring both the promise of digital transformation and the challenges of training, infrastructure, and governance in the region.

Despite widespread optimism about the benefits of digital transformation, actual adoption remains limited, with fewer participants expressed satisfaction with the extent to which their institutions actively incorporate advanced technologies. While tools such as EHRs are gaining traction, more advanced technologies like AI, VR, and robotics are underused in clinical and research workflows. The modest adoption score reflects partial implementation of digital systems rather than systemic integration. This may be explained by the infrastructural gaps reported by over half the participants, along with the lack of central initiatives to guide regional digital transformation efforts. Other barriers to adoption faced by healthcare institutions in the MENA region include infrastructural, financial, and educational challenges [8,10,30–33]. These findings echo SDG 9 (Industry, Innovation, and Infrastructure), calling for targeted investments to enhance accessibility and technological equity [34].

In contrast to the moderate levels of digital adoption we observed in the MENA region, recent global reports indicate a considerable digital health gap. While countries such as the UAE and Saudi Arabia show consumer engagement levels comparable to North America and Europe—with near-universal internet access and rapidly growing digital health markets—regional digital inclusion overall remains below global averages [35,36], and health systems continue to struggle with fragmentation and suboptimal deployment [9,10]. This pattern reflects broader global trends: only half of European and Central Asian countries have digital health literacy policies, and provider digital maturity scores in Europe and the U.S. remain modest [14,37]. Meanwhile, in MENA, digital inclusion remains below global averages, and regional health systems still lag and score below global regions in metrics like affordability, accessibility, and ability [14].

Evidence from regional and global studies highlights that strengthening infrastructure, embedding ethics into training, and building robust regulatory frameworks are essential to ensure digital health transformation is not only technologically feasible but also equitable, ethical, and sustainable [10,38–41]. Our findings therefore underscore the importance of context-specific strategies tailored to regional disparities in digital infrastructure, policy readiness, and capacity building.

Urban residence, digital proficiency, and formal training were significantly associated with higher adoption scores (*p* = 0.004), underscoring the presence of digital infrastructure disparities and the observed urban-rural digital divide. This finding aligns with concerns raised by the World Health Organization, which cautions that the benefits of AI may disproportionately favor wealthier populations, marginalizing underserved or conflict-affected communities [42]. Therefore, multidisciplinary and interdisciplinary collaboration is essential to ensure digital tool designs are equitable and representative to address the needs and concerns of different stakeholders, avoiding the risks of algorithmic bias, exclusion of vulnerable groups, or revealing potential conflicts of interest [43,44].

Although detailed country-level subgroup analyses were beyond the scope of this study, it remains crucial to acknowledge the contextual diversity across the MENA region. High-resource countries—such as the UAE and Saudi Arabia—benefit from robust digital infrastructure, substantial investment, and strategic governance frameworks; for example, Saudi Arabia has driven massive healthcare digitization under Vision 2030, and the UAE records a majority of healthcare sector investment activity in the Gulf [45]. In contrast, conflict-affected settings like Yemen continue to face significant barriers, including damaged infrastructure, governance gaps, and severely fragmented health systems [38].

Descriptive trends in our data also suggest variation in stakeholder responses across this spectrum—from advanced digital health policies in nations like the UAE and Saudi Arabia to the more constrained settings in Sudan. Future research should explicitly stratify analyses by sub-regions or digital maturity, enabling a more nuanced understanding of context-specific challenges and the identification of success stories that can be scaled or adapted across diverse settings.

The predominance of millennials in the sample (mean age 31.1 years) is noteworthy, given this generation is often characterized by their familiarity with technology and social media fluency [46]. As “digital natives”, millennials may be more open to engaging with digital health innovations, but they also bring specific expectations around transparency, authenticity, and accessibility in healthcare delivery. Their distinct health concerns, shaped by economic instability and rising mental health awareness, further inform the ethical frameworks within which digital health tools must be developed [47,48]. Despite this, the reported lack of formal training, even among digitally proficient participants, calls attention to the need for ongoing structured education that equips healthcare workers with the skills needed to integrate digital tools into the research and practice effectively.

Moreover, aligning with SDG 4 (quality education), there is a strong need to provide training on the development, deployment, and practical clinical application of digital technologies, particularly AI [49]. Telemedicine, for example, has the potential to transform healthcare access, especially in remote and underserved areas [50], while wearable health devices and blockchain technology offer promising solutions for real-time monitoring of chronic conditions and secure data management [51,52]. Nonetheless, unique barriers such as weak infrastructure, limited regulatory oversight, and disparities in digital literacy—among both providers and patients—continue to impede widespread progress of these technologies in the MENA region [53].

Our findings emphasize the necessity for longitudinal, ethics-integrated education for healthcare professionals. This is particularly important in equipping future public health leaders to address the ethical and logistical challenges posed by digital transformation. While digital tools such as AI and mHealth applications can be especially valuable in managing non-communicable and infectious diseases [54,55], their effectiveness depends on legislative clarity, robust data governance, and institutional readiness [56]. Many existing healthcare and research institutions in the MENA region lack the infrastructure to support advanced digital tools, leading to fragmented systems and inefficiencies [9,10].

Despite the limited institutional preparedness, attitudinal scores and ethical consideration scores were generally favorable and optimistic, with most participants agreeing that digital health technologies can enhance future clinical decision-making and health outcomes. However, the mismatch between positive attitudes and inadequate training signals a critical gap in capacity building, as only one-fifth felt adequately trained, and half reported dissatisfaction with the current level of digital transformation in their institutions. This gap suggests that while stakeholders acknowledge the promise of digital health, practical barriers—training, support, and governance—remain unresolved. Notably, respondents with advanced degrees (e.g., PhD holders) and digital proficiency were more likely to hold positive attitudes, suggesting the need for tailored, hands-on training programs that accommodate diverse technical backgrounds [34,57]. While participants were generally well-educated, the lack of formal training may hinder the effective adoption of digital tools, which are becoming increasingly complex, particularly with advancements in AI, big data analytics, and wearable health devices [11,58].

Interestingly, logistic regression revealed a paradox: participants who had received formal training were less likely to report positive attitudes and more likely to express ethical concerns. One possible explanation may be that training exposes professionals to the practical risks of digital health, including data privacy challenges and governance gaps, thereby increasing awareness of potential harms. For instance, a study found that healthcare staff who completed IT security training were 4.2 times more likely to correctly respond to a potential breach than those who did not, demonstrating how training elevates recognition of risk scenarios and the actions needed to mitigate them [59]. This suggests that greater exposure to the complexities of digital health fosters critical evaluation of risks such as algorithmic bias, threats to autonomy, and data misuse. Training may thus enhance competence while also sharpening recognition of the ethical and regulatory safeguards required for safe implementation.

It may further reflect limitations in the design of current programs, which often neglect the socio-political nuances and infrastructural realities of the MENA region. These issues are particularly salient in fragile contexts, where regulatory systems are underdeveloped. This underscores the need for balanced digital health education to incorporate ethical realism alongside technical competence and for robust stakeholder collaboration to ensure trust, accountability, and transparency, in line with SDG 16 (peace, justice, and strong institutions).

The study revealed a strong ethical awareness among participants, with over two-thirds advocating for core bioethical principles, such as privacy, autonomy, and justice, in guiding the development and deployment of digital health technologies. Yet, only a third reported familiarity with regulatory guidelines for digital health in clinical research, reflecting a lack of harmonized regional frameworks in the MENA region, where regulatory structures are still evolving [60,61]. Concerns about informed consent, data protection, and algorithmic accountability were prevalent, particularly among female participants and those with longer research experience. This highlights an urgent need to integrate ethics education into digital health curricula and to establish region-specific regulatory oversight mechanisms.

More than two-thirds called for continuous oversight and stakeholder education. Concerns about health inequities, especially during pandemics, and the ethical implications of algorithmic decision-making were common. Vulnerable populations, such as refugees or those in conflict-affected areas, may be left behind in this digital revolution. So, as the MENA region continues to embrace digital transformation, stakeholders must prioritize equitable access to health technologies to avoid exacerbating existing disparities. Ethical considerations surrounding governance, data security, and patient privacy become paramount in environments marked by distrust in unstable governmental systems [18,62]. These findings resonate with SDG 3 (good health and well-being) and SDG 16, reinforcing the need for national regulatory frameworks that prioritize transparency, patient rights, and data governance.

Importantly, over 60% of participants reported using wellness apps, yet only 33% were aware of any regulatory guidelines for digital tools in research. This suggests a dangerous mismatch between usage and governance as these apps collect, store, and share vast amounts of sensitive health information. Concepts such as “data colonialism,” wherein health data from low-income regions is extracted without ethical safeguards, become especially relevant in the absence of strong institutional protections [42]. Addressing disruptive technologies issues requires a multi-faceted approach involving targeted educational health informatics initiatives, technical support, key performance metrics reporting, impartiality, and fostering a culture of innovation within different healthcare systems and research settings [63,64].

Multivariate analyses identified several predictors of favorable attitudes and ethical considerations. Digital proficiency, prior research experience, and familiarity with digital health concepts were all positively associated with better scores across key domains. Interestingly, formal training in digital health was inversely associated with positive attitudes and ethical confidence, possibly reflecting dissatisfaction with the quality/applicability of existing training programs or failure to adequately address regional needs. This finding indicates that mere exposure to training is insufficient without high-quality, context-relevant instruction that addresses both technical and ethical dimensions, and a balanced approach that highlights both the potential benefits and ethical complexities of digital health.

Subgroup analyses revealed key disparities. Participants with PhDs, higher English and digital proficiency, and extensive research experience were significantly more likely to report ethical and optimistic views. Gender differences also emerged, with female participants demonstrating more ethical caution—echoing findings from global literature on gendered perspectives in healthcare ethics [65,66]. These insights suggest that capacity-building efforts should be stratified to address educational and experiential gaps within healthcare workforces.

The region’s successful digital transformation will depend on international collaboration and the establishment of robust, adaptable legal frameworks, as envisioned by SDG 17 (partnerships for the goals) [9]. Regulatory responses must keep pace with rapid technological changes to protect patient autonomy and prevent misuse. User-centered design, especially for populations with disabilities or limited digital literacy, is critical for inclusive adoption.

### Implications *for* policy and practice

To realize the potential of digital health and move toward an ethically grounded digital transformation in MENA, multi-level interventions are required. These include: (1) strengthening institutional and infrastructural readiness by bridging the rural-urban divide and ensuring interoperability across platforms; (2) offering interdisciplinary training programs in both advanced digital competencies and bioethics in clinical settings to ensure preparedness; (3) improving awareness of regional initiatives; and (4) creating adaptive governance frameworks that are transparent and responsive to local cultural and ethical contexts. The significant role of regulatory monitoring, as highlighted by participants, points to the need for stronger government leadership in coordinating digital health efforts and resisting undue influence from commercial technology stakeholders. Regulatory frameworks must be developed or strengthened to address data privacy, ethical use, and industry influence. Blockchain, smart contracts, and decentralized technologies may offer useful tools in high-risk contexts. Ultimately, policymakers, researchers, technologists, and healthcare providers must collaborate in the co-development of context-sensitive digital health strategies. Public engagement efforts can help foster trust and enhance the user experience, particularly in conflict-affected or underserved communities. Financial incentives should encourage stakeholders to adopt digital health technologies, while international support can help bridge gaps left by unstable governments.

### Limitations and future directions

This study is among the first to offer a regionally representative assessment of digital health attitudes and ethics in 20 MENA countries. Its strengths include a large sample size and the integration of multiple dimensions into a unified analysis. However, its cross-sectional design limits inference to associations and does not permit causal conclusions. Reliance on self-reported data may also introduce selection bias or social desirability, particularly given the disproportionate representation from Jordan (39.2% of respondents). Moreover, the absence of open-ended responses restricted our ability to capture the reasons behind some of the observed attitudes, limiting qualitative depth. Generalizability is further constrained by contextual diversity within the region, where digital health adoption varies between high-resource settings and countries affected by conflict or instability. Additionally, the sample’s diversity and the study’s focus on various encounters suggest that the results should be interpreted cautiously. Future research should incorporate longitudinal and mixed-method approaches, assess the actual outcomes of digital health interventions, and explore how legal, religious, and cultural contexts shape ethical perceptions and governance. Comparative studies across regions with different levels of digital maturity could provide further valuable insights for country-specific policy frameworks and best practices.

## Conclusion

Digital transformation in healthcare across the MENA region stands at a critical juncture, characterized by high aspirations but tempered by systemic constraints and ethical ambiguities/uncertainties as viewed by healthcare professionals and researchers. This study highlights that while attitudes toward digital health are mainly positive, gaps in infrastructure, governance, and training remain evident and hinder the effective implementation of digital health initiatives. Addressing these challenges through inclusive policy frameworks, sustained regulatory support, strategic investments, and robust education programs are required. Prioritizing ethical governance and cross-sector collaboration will be essential to unlock the full potential of digital health. In doing so, stakeholders in the region can ensure a transformation that is not only technologically advanced/effective but also trustworthy, equitable, ethical, and sustainable. By equipping the next generation of healthcare professionals and researchers with the necessary skills and ethical frameworks, the region can achieve a transformation that strengthens both clinical outcomes and research practices.

## Supporting information

S1 Appendix Survey questionnaire. Full survey instrument used in the study, including sociodemographic items, digital health adoption, attitudes, and ethical considerations.
(DOCX)

S2 Appendix Supplementary tables. S1-S8 Tables, presenting additional descriptive statistics, subgroup analyses, and regression results supporting the main findings.
(DOCX)

S1 Dataset Raw study data. Unprocessed dataset containing the study’s findings. (XLSX)
