# Supplementary material for: Perceived clinical and ethical impact of digital transformation in healthcare and research: A survey in the MENA region
Source: PLoS One. 2025 Dec 2;20(12):e0336618. doi: 10.1371/journal.pone.0336618 (PMC12671818; doi:10.1371/journal.pone.0336618)
Supplement: S1 Appendix — Full survey instrument used in the study, including sociodemographic items, digital health adoption, attitudes, and ethical considerations. (DOCX) [file pone.0336618.s001.docx]

**‏‏Questionnaire**

**The Clinical and Ethical Impact of Digital Transformation in Health Research: A Survey in MENA Region**

Welcome to our research survey on the ethical considerations and perspectives regarding the digital transformation for better health and well-being. This survey is a part of a research project aimed at better understanding the issues related to digital health and biomedical ethics.

Before you proceed with the survey, we would like to inform you about the nature of this research and seek your informed consent. Please read the following information carefully:

- This survey is designed to collect data on your opinions, concerns, and perspectives related to digital transformation. Your input will contribute to a better understanding of the ethical implications of digital health adoption in research and its potential impact on patient care.
- Your participation in this survey is entirely voluntary. You are under no obligation to complete the survey, and you may withdraw at any time without consequence.
- All responses will be kept confidential. Your individual responses will not be shared or associated with your personal identity. Data will be analyzed and reported in aggregate form to ensure anonymity.
- The survey will take approximately 10-15 minutes to complete.
- If you have any questions or concerns about this survey or your participation, please contact [hehassan23@ph.just.edu.jo](mailto:hehassan23@ph.just.edu.jo).
- This survey can only be completed once. So, if you have already completed this survey, please do not proceed.
- Please try to give as honest answers as possible. Do not Google answers you do not know or guess. Just choose what you know, and if you do not know the answer, simply choose “I don’t know” or “Neutral”.

Thank you for your time and valuable input.

Do you agree to participate in this survey?

- Yes

**Section 1: Sociodemographic** **Information**

1. Which country in the MENA region are you associated with?

- Randomized Country List Options

2. Geographic Location:

- Urban Setting
- Rural Setting

3. Gender:

- Male
- Female

4. Age (Years):

- Filling by English number: ________

5. Profession: What is your primary role in the healthcare sector?

- Physician
- Pharmacist
- Nurse
- Allied health professional (laboratories, physical therapy, nutrition, etc.)
- Medical Researcher /Academic
- Technology developer /IT professional
- Other: _________

6. How do you rate your income level (according to your country's wage system)?

- Lower income
- Middle income
- Upper income

7. Educational Background (highest level of education completed):

- Bachelor degree
- Master degree
- Doctor of Philosophy (PhD)

8. Employment Setting/ Work Environment:

- Unemployed
- Student or Internship/trainee
- Hospital or clinic
- Private business (pharmacy, laboratory, etc.)
- Academic or research institution
- Entrepreneurial company or industry
- Government agency
- Non-governmental organization (NGO)
- Other: __________

9. Professional Sector Affiliation:

- Public (governmental)
- Private

10. How would you rate your proficiency in English?

- Fluent
- Proficient
- Basic

11. How proficient are you with using digital technologies?

- Very high
- High
- Neutral
- Low
- Very low

12. Have you received any formal training or education on digital health topics?

- Yes
- No

13. Years of experience in research:

- None/ Not applicable
- Less than 1 year
- 1-5 years
- 6-10 years
- More than 10 years

**Section 2: Digital Health Adoption and Transformation**

1. How familiar are you with: (randomized order)

|  | **Not familiar at all** | **Not familiar** | **Neutral** | **Familiar** | **Very familiar** |
| --- | --- | --- | --- | --- | --- |
| The concept of digital transformation in healthcare |  |  |  |  |  |
| Digital health concepts and terminology |  |  |  |  |  |
| The use of digital health technologies (eHealth, mHealth) in research settings |  |  |  |  |  |

2. What specific digital health technologies are currently prevalent in the healthcare sector in your country? (Select all that apply) (randomized order)

- Electronic Health Records (EHR)
- Telehealth services/Telemedicine
- E-Pharmacy services
- Data analytics and Artificial Intelligence (AI)
- Wearable health devices
- Mobile Health (mHealth) applications
- Digital training and education
- Public health campaigns through social media
- Virtual Reality (VR)
- Robotics

3. How satisfied were you with the current level of digital health adoption in your institution (*i.e.,* the use of wearable devices, remote monitoring, or AI for managing in your healthcare system)?

1 2 3 4 5

Not satisfied at all Very satisfied

4. My organization actively incorporates emerging technologies, such as voice interfaces, augmented reality, AI, cloud storage, and blockchain, etc., into daily work routines as it has a well-defined process for implementing and collaborating on digital health solutions.

1 2 3 4 5

Strongly disagree Strongly agree

5. Answer the following questions (randomized order):

| **Question** | **Yes** | **No** |
| --- | --- | --- |
| Are you aware of any central initiatives or programs aimed at driving digital transformation in healthcare and research in your region? |  |  |
| Do you believe that your region has the necessary infrastructure to support digital transformation in healthcare and research? |  |  |
| Have you participated in clinical research that utilized digital solutions? |  |  |
| Do you think there is enough research evidence available to support the adoption of digital health technologies in the MENA region? |  |  |
| Have you ever downloaded and used a health or wellness mobile app on your smartphone? |  |  |
| Are you aware of any regulatory guidelines or best practices related to the use of digital tools in clinical trials? |  |  |

**Section 3: Attitudes towards Digital Health in Research**

1. How do you perceive the impact of digital health technologies on research practices in your region?

| 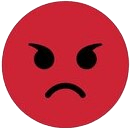 | 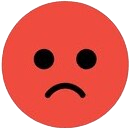 | 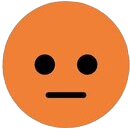 | 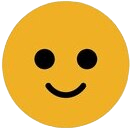 | 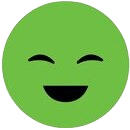 |
| --- | --- | --- | --- | --- |
| Very negative | Negative | Neutral | Positive | Very positive |

2. Please rate the following statements on a 5-point Likert scale (randomized order):

| **Statement** | **Strongly Disagree** | **Disagree** | **Neutral** | **Agree** | **Strongly Agree** |
| --- | --- | --- | --- | --- | --- |
| Digital technologies contribute to better health and well-being in the future |  |  |  |  |  |
| Using digital health in my specialty could improve clinical decision-making |  |  |  |  |  |
| I am optimistic about digital health technologies in improving public health outcomes |  |  |  |  |  |
| Digital health technologies will reduce the financial costs associated with my role |  |  |  |  |  |
| Digital health technologies may take over part of my role as a healthcare professional in the future |  |  |  |  |  |
| I have been adequately trained to use digital health that is specific to my role |  |  |  |  |  |
| The effectiveness of digital health technologies heavily relies on the quality of their underlying algorithms |  |  |  |  |  |
| Digital health interventions could effectively address the higher prevalence of the stigma surrounding mental health issues in our communities |  |  |  |  |  |
| Digital solutions enhance efficiency and effectiveness in clinical research, particularly increasing participation from minorities in remote communities |  |  |  |  |  |
| I anticipate common challenges when using digital solutions in healthcare and clinical research, such as cultural resistance to change or digitization |  |  |  |  |  |

**Section 4: Ethical Considerations of Digital Transformation**

1. How do you evaluate the application of the current ethical framework for digital health technologies in human research?

| 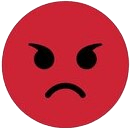 | 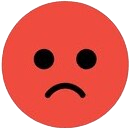 | 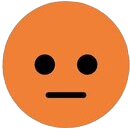 | 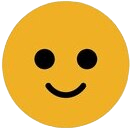 | 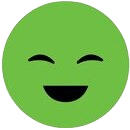 |
| --- | --- | --- | --- | --- |
| Very negative | Negative | Neutral | Positive | Very positive |

2. Please rate the following statements on a 5-point Likert scale (randomized order):

| **Statement** | **Strongly Disagree** | **Disagree** | **Neutral** | **Agree** | **Strongly Agree** |
| --- | --- | --- | --- | --- | --- |
| I believe that digital health technologies should prioritize principles such as respect for autonomy, beneficence, non-maleficence, and justice to provide a comprehensive ethical decision-making process |  |  |  |  |  |
| I am concerned about ensuring proper informed consent procedures in humanitarian research conducted using digital tools |  |  |  |  |  |
| I think that ensuring privacy, confidentiality, security, and fairness in the use of digital health technologies is crucial, especially in clinical research |  |  |  |  |  |
| I am concerned that even the effective use of medical and personal data in digital healthcare will raise ethical concerns, particularly regarding the protection of the rights and identity of research subjects |  |  |  |  |  |
| I believe that access to digital health care should be considered a basic right, and efforts must be made to ensure equitable access to necessary infrastructure |  |  |  |  |  |
| I am concerned about the potential exacerbation of health inequities by digital technologies, especially in the context of pandemics |  |  |  |  |  |
| I believe that stakeholders in the digital health ecosystem should have a better understanding of bioethical concepts and their application in practice |  |  |  |  |  |
| I am aware of the ethical issues, challenges, and harms associated with digital health technologies |  |  |  |  |  |
| Governments should actively resist undue influence from large technology corporations in shaping health-related policies, ensuring public interests are prioritized |  |  |  |  |  |
| There is a need for greater awareness and consideration of the social and ethical implications of technological advancements in health research |  |  |  |  |  |
| The quality and functionality of digital healthcare systems depend only on technical aspects and not on their effective adoption and ethical values |  |  |  |  |  |
| Personalized medicine should be accessible to all individuals based on evidence that reflects their lived experience and biology, treating them equally according to their needs |  |  |  |  |  |
| I am aware of various approaches, including Islamic religious, socio-cultural, and political factors, involved in the transparent and interactive assessment of health technology and innovation |  |  |  |  |  |
| Regular monitoring of digital health systems is essential to identify problems and ensure adherence to specifications |  |  |  |  |  |
| Establishing a no-blame reporting culture is crucial for identifying and rectifying problems in digital health systems, ensuring optimal trust and utilization by users |  |  |  |  |  |

3. What measure do you think is the most important to be taken to ensure the ethical use of digital tools in clinical research? (randomized order)

- Strengthening data security measures
- Providing clear informed consent processes
- Regular monitoring and oversight by regulatory bodies
- Ensuring transparency in data handling practices and policies
- Participant education on data sharing practices
- Tailored support for older adults
- Financial assistance for acquiring digital devices
