# Supplementary material for: Perceived clinical and ethical impact of digital transformation in healthcare and research: A survey in the MENA region
Source: PLoS One. 2025 Dec 2;20(12):e0336618. doi: 10.1371/journal.pone.0336618 (PMC12671818; doi:10.1371/journal.pone.0336618)
Supplement: S2 Appendix — S1-S8 Tables, presenting additional descriptive statistics, subgroup analyses, and regression results supporting the main findings. (DOCX) [file pone.0336618.s002.docx]

**Supporting information**

**Table S1:** Additional details on the sociodemographic characteristics of the study participants.

| **Item** | ***n* (%)** |
| --- | --- |
| **Country** | |
| Algeria | 6 (0.6) |
| Bahrain | 8 (0.8) |
| Egypt | 148 (14.9) |
| Iraq | 85 (8.6) |
| Jordan | 388 (39.2) |
| Kuwait | 12 (1.2) |
| Lebanon | 15 (1.5) |
| Libya | 17 (1.7) |
| Mauritania | 2 (0.2) |
| Morocco | 7 (0.7) |
| Oman | 9 (0.9) |
| Palestine | 15 (1.5) |
| Qatar | 9 (0.9) |
| Saudi Arabia | 80 (8.1) |
| Somalia | 1 (0.1) |
| Sudan | 103 (10.4) |
| Syria | 17 (1.7) |
| Tunisia | 2 (0.2) |
| United Arab Emirates | 47 (4.7) |
| Yemen | 19 (1.9) |
| **What is your primary role in the healthcare sector?** | |
| Physician | 276 (27.9) |
| Pharmacist | 194 (19.6) |
| Nurse | 69 (7.0) |
| Allied health professional (laboratories, physical therapy, nutrition, etc.) | 138 (13.9) |
| Medical Researcher /Academic | 226 (22.8) |
| Technology developer /IT professional | 44(4.4) |
| Others^*^ | 43 (4.3) |

^*^ This group includes quality assurance, medical informatics, bioethics, medical law, medical fiqh, psychiatric roles, medical military, and medical media/marketing specialists.

**Table S2:** Familiarity and satisfaction with digital health adoption and transformation (n=990).

| **Item** | ***n* (%)** | | | | | ***m* ± SD** |
| --- | --- | --- | --- | --- | --- | --- |
| **Familiarity** | | | | | | |
| **How familiar are you with** | **Very familiar** | **Familiar** | **Neutral** | **Not familiar** | **Not familiar at all** | ***m* ± SD** |
| The concept of digital transformation in healthcare | 46 (4.6) | 342 (34.5) | 282 (28.5) | 262 (26.5) | 58 (5.9) | 3.1 ± 1.0 |
| Digital health concepts and terminology | 31 (3.1) | 294 (29.7) | 343 (34.6) | 262 (26.5) | 60 (6.1) | 3.0 ± 1.0 |
| The use of digital health technologies (eHealth, mHealth) in research settings | 42 (4.2) | 315 (31.8) | 291 (29.4) | 278 (28.1) | 64 (6.5) | 3.0 ± 1.0 |
| **Satisfaction** | | | | | | |
| **How satisfied were you with** | **Very satisfied** | **Satisfied** | **Neutral** | **Not satisfied** | **Not satisfied at all** | ***m* ± SD** |
| The current level of digital health adoption in your institution (*i.e.*, the use of wearable devices, remote monitoring, or AI for managing in your healthcare system) | 66 (6.7) | 125 (12.6) | 304 (30.7) | 259 (26.2) | 236 (23.8) | 2.5 ± 1.2 |
| Your organization actively incorporates emerging technologies (such as voice interfaces, augmented reality, AI, cloud storage, blockchain, etc.) into daily work routines as it has a well-defined process for implementing and collaborating on digital health solutions | 87 (8.8) | 144 (14.5) | 312 (31.5) | 222 (22.4) | 225 (22.7) | 2.6 ± 1.2 |

**Table S3:** Awareness and engagement in digital health initiatives (n=990).

| **Question** | ***n* (%)** | | ***m* ± SD** |
| --- | --- | --- | --- |
|  | **Yes** | **No** |  |
| Are you aware of any central initiatives or programs aimed at driving digital transformation in healthcare and research in your region? | 292 (29.5) | 698 (70.5) | 0.3 ± 0.5 |
| Do you believe that your region has the necessary infrastructure to support digital transformation in healthcare and research? | 452 (45.7) | 538 (54.3) | 0.5 ± 0.5 |
| Have you participated in clinical research that utilized digital solutions? | 177 (17.9) | 813 (82.1) | 0.2 ± 0.4 |
| Do you think there is enough research evidence available to support the adoption of digital health technologies in the MENA region? | 434 (43.8) | 556 (56.2) | 0.4 ± 0.5 |
| Have you ever downloaded and used a health or wellness mobile app on your smartphone? | 610 (61.6) | 380 (38.4) | 0.6 ± 0.5 |
| Are you aware of any regulatory guidelines or best practices related to the use of digital tools in clinical trials? | 327 (33.0) | 663 (67.0) | 0.3 ± 0.5 |

**Table S4:** Summary of normalized scores for the total research study sections: adoption, attitude, and ethical considerations in digital health technologies (n=990).

| **Total score categories^*^** | ***m* ± SD** |
| --- | --- |
| 1. Digital health adoption and transformation score^**^ | 0.41 ± 0.16 |
| 1. Integration magnitude (familiarity + satisfaction) score | 0.57 ± 0.15 |
| - Total familiarity score | 0.60 ± 0.18 |
| - Total satisfaction score | 0.52 ± 0.21 |
| 1. Number of the used digital health technologies score | 0.29 ± 0.21 |
| 1. Awareness in initiatives and engagement score | 0.39 ± 0.28 |
| 1. Attitudes towards digital health in research score | 0.69 ± 0.13 |
| 1. Ethical considerations in digital health score | 0.70 ± 0.12 |

^*^ Note: Scores from each category have been normalized (*i.e.*, the sum of all responses divided by the maximum possible score) to a common scale ranging from 0 to 1. Higher normalized scores reflect more positive responses or greater levels of familiarity, satisfaction, adoption, attitudes, or ethical considerations.

^**^ Specifically, adoption refers to the initial acceptance and utilization of these technologies, transformation denotes broader changes in healthcare practices driven by these tools, and integration evaluates the incorporation of these tools into existing workflows.

**Table S5:** Parameters affecting the normalized research study sections scores (n=990).

| **Variable** | | **Adoption** | | **Attitudes** | | **Ethics** | |
| --- | --- | --- | --- | --- | --- | --- | --- |
| **Category** | **Subcategory** | ***m* ± SD** | ***p*-value^*^** | ***m* ± SD** | ***p*-value^*^** | ***m* ± SD** | ***p*-value^*^** |
| Geographic location | Urban | 0.42 ± 0.16 | **0.004** | 0.70 ± 0.13 | 0.519 | 0.70 ± 0.13 | 0.290 |
|  | Rural | 0.38 ± 0.15 |  | 0.69 ± 0.12 |  | 0.69 ± 0.12 |  |
| Gender | Male | 0.42 ± 0.17 | 0.181 | 0.70 ± 0.14 | 0.624 | 0.69 ± 0.14 | 0.398 |
|  | Female | 0.41 ± 0.15 |  | 0.70 ± 0.12 |  | 0.70 ± 0.12 |  |
| Profession | Healthcare providers | 0.41 ± 0.16 | 0.306 | 0.69 ± 0.12 | 0.831 | 0.70 ± 0.12 | 0.473 |
|  | Medical researchers or academic | 0.41 ± 0.17 |  | 0.69 ± 0.15 |  | 0.70 ± 0.15 |  |
|  | Others | 0.44 ± 0.17 |  | 0.69 ± 0.13 |  | 0.71 ± 0.12 |  |
| Income level | Lower income | 0.37 ± 0.15 | **<0.001** | 0.69 ± 0.12 | 0.819 | 0.70 ± 0.12 | 0.291 |
|  | Middle income | 0.43 ± 0.16 |  | 0.69 ± 0.12 |  | 0.70 ± 0.12 |  |
|  | Upper income | 0.44 ± 0.17 |  | 0.68 ± 0.14 |  | 0.68 ± 0.13 |  |
| Educational background | Bachelor degree | 0.40 ± 0.15 | **0.004** | 0.68 ± 0.12 | **0.007** | 0.69 ± 0.12 | **0.041** |
|  | Master degree | 0.44 ± 0.17 |  | 0.70 ± 0.14 |  | 0.71 ± 0.13 |  |
|  | PhD | 0.43 ± 0.17 |  | 0.71 ± 0.14 |  | 0.72 ± 0.14 |  |
| Employment setting | Unemployed | 0.40 ± 0.16 | 0.573 | 0.70 ± 0.12 | 0.182 | 0.72 ± 0.12 | 0.142 |
|  | Internship | 0.42 ± 0.16 |  | 0.68 ± 0.11 |  | 0.69 ± 0.11 |  |
|  | Hospital or clinic | 0.40 ± 0.16 |  | 0.69 ± 0.13 |  | 0.69 ± 0.13 |  |
|  | Private business | 0.41 ± 0.17 |  | 0.68 ± 0.14 |  | 0.69 ± 0.15 |  |
|  | Academic or research institution | 0.42 ± 0.17 |  | 0.71 ± 0.13 |  | 0.72 ± 0.13 |  |
|  | Entrepreneurial company or industry | 0.44 ± 0.14 |  | 0.69 ± 0.11 |  | 0.71 ± 0.10 |  |
|  | Government agency | 0.45 ± 0.15 |  | 0.71 ± 0.16 |  | 0.69 ± 0.16 |  |
|  | Non-governmental organization (NGO) | 0.43 ± 0.14 |  | 0.66 ± 0.15 |  | 0.67 ± 0.12 |  |
| Professional sector affiliation | Not applicable | 0.41 ± 0.15 | 0.981 | 0.67 ± 0.09 | **0.002** | 0.68 ± 0.08 | **0.019** |
|  | Public (governmental) | 0.41 ± 0.16 |  | 0.70 ± 0.13 |  | 0.71 ± 0.13 |  |
|  | Private | 0.41 ± 0.16 |  | 0.68 ± 0.14 |  | 0.69 ± 0.14 |  |
| English proficiency | Fluent | 0.46 ± 0.17 | **<0.001** | 0.71 ± 0.12 | **0.006** | 0.71 ± 0.12 | 0.136 |
|  | Proficient | 0.40 ± 0.16 |  | 0.69 ± 0.12 |  | 0.70 ± 0.12 |  |
|  | Basic | 0.39 ± 0.15 |  | 0.67 ± 0.14 |  | 0.69 ± 0.14 |  |
| Digital technologies utilization proficiency | Very high | 0.52 ± 0.17 | **<0.001** | 0.72 ± 0.16 | **<0.001** | 0.70 ± 0.15 | **<0.001** |
|  | High | 0.43 ± 0.16 |  | 0.71 ± 0.11 |  | 0.72 ± 0.11 |  |
|  | Neutral | 0.37 ± 0.14 |  | 0.67 ± 0.12 |  | 0.68 ± 0.12 |  |
|  | Low | 0.36 ± 0.14 |  | 0.65 ± 0.14 |  | 0.67 ± 0.14 |  |
|  | Very low | 0.28 ± 0.10 |  | 0.64 ± 0.06 |  | 0.65 ± 0.07 |  |
| Received any formal training or education on digital health topics | Yes | 0.51 ± 0.16 | **<0.001** | 0.70 ± 0.14 | 0.162 | 0.69 ± 0.14 | 0.370 |
|  | No | 0.39 ± 0.15 |  | 0.69 ± 0.12 |  | 0.70 ± 0.12 |  |
| Years of experience in research | None/ Not applicable | 0.38 ± 0.15 | **<0.001** | 0.68 ± 0.12 | **0.004** | 0.69 ± 0.12 | **0.010** |
|  | Less than 1 year | 0.14 ± 0.15 |  | 0.69 ± 0.11 |  | 0.70 ± 0.12 |  |
|  | 1-5 years | 0.44 ± 0.16 |  | 0.69 ± 0.13 |  | 0.69 ± 0.12 |  |
|  | 6-10 years | 0.45 ± 0.16 |  | 0.73 ± 0.11 |  | 0.74 ± 0.10 |  |
|  | More than 10 years | 0.45 ± 0.19 |  | 0.71 ± 0.15 |  | 0.71 ± 0.15 |  |

^*^ A *p*-value of less than 0.05 indicates statistical significance, calculated by independent *t*-test or one-way ANOVA when appropriate.

**Table S6:** Summary of the overall multiple linear regression model analysis results (n=990).

| **Dependent variable** | **Significant predictors** | **Pearson correlation coefficient (*r*)** | **Standardized coefficient (β)** | **R^2^** | **F-statistic** | ***p*-value^*^** |
| --- | --- | --- | --- | --- | --- | --- |
| Attitudes towards digital health in research score | Digital health adoption and transformation score | 0.316 | 0.151 | 0.586 | 699.5 | **<0.001** |
|  | Ethical considerations in digital health score | 0.752 | 0.717 |  |  |  |
| Ethical considerations in digital health score | Digital health adoption and transformation score | 0.231 | -0.007 | 0.565 | 640.8 | **<0.001** |
|  | Attitudes towards digital health in research score | 0.752 | 0.754 |  |  |  |

^*^ A *p*-value of less than 0.05 indicates statistical significance, calculated by ANOVA’s linear regression.

**Table S7:** Logistic regression analysis results for the prediction of positive attitude towards digital health transformation in clinical research (n=990).

| **Predictor** | **Sub-category** | **OR** | **95% CI. for OR** | | ***p*-value^*^** |
| --- | --- | --- | --- | --- | --- |
|  |  |  | **Lower** | **Upper** |  |
| Gender | Male | 0.831 | 0.611 | 1.129 | 0.237 |
|  | Female (REF) | - | - | - | 0 |
| Age (Years) | | 1.018 | 0.996 | 1.040 | 0.111 |
| Profession | Healthcare providers | 1.019 | 0.620 | 1.675 | 0.940 |
|  | Medical researchers or academic | 1.093 | 0.605 | 1.977 | 0.768 |
|  | Others (REF) | - | - | - | 0 |
| Income level | Lower income | 0.761 | 0.436 | 1.327 | 0.336 |
|  | Middle income | 0.715 | 0.426 | 1.198 | 0.202 |
|  | Upper income (REF) | - | - | - | 0 |
| Educational background | Bachelor degree | 0.987 | 0.525 | 1.856 | 0.967 |
|  | Master degree | 0.813 | 0.465 | 1.421 | 0.467 |
|  | PhD (REF) | - | - | - | 0 |
| Professional sector affiliation | Unemployed (REF) | - | - | - | 0 |
|  | Student or Internship | 0.889 | 0.533 | 1.483 | 0.653 |
|  | Hospital or clinic | 0.840 | 0.481 | 1.468 | 0.540 |
|  | Private business | 0.793 | 0.417 | 1.508 | 0.479 |
|  | Academic or research institution | 1.193 | 0.627 | 2.270 | 0.591 |
|  | Entrepreneurial company or industry | 0.599 | 0.239 | 1.499 | 0.273 |
|  | Government agency | 0.906 | 0.427 | 1.920 | 0.796 |
|  | NGO | 0.810 | 0.271 | 2.416 | 0.705 |
| Professional sector affiliation | Not applicable (REF) | - | - | - | 0 |
|  | Public | 1.147 | 0.737 | 1.787 | 0.543 |
|  | Private | 0.874 | 0.546 | 1.401 | 0.577 |
| English proficiency | Fluent (REF) | - | - | - | 0 |
|  | Proficient | 0.880 | 0.621 | 1.248 | 0.473 |
|  | Basic | 0.787 | 0.521 | 1.190 | 0.256 |
| Digital technologies utilization proficiency | Very high | 0.740 | 0.212 | 2.586 | 0.637 |
|  | High | 0.738 | 0.393 | 1.386 | 0.345 |
|  | Neutral | 0.737 | 0.453 | 1.199 | 0.219 |
|  | Low | 1.054 | 0.673 | 1.649 | 0.819 |
|  | Very low (REF) | - | - | - | 0 |
| Received any formal training or education on digital health topics | Yes | 0.547 | 0.378 | 0.792 | **0.001** |
|  | No (REF) | - | - | - | 0 |
| Years of experience in research | None (REF) | - | - | - | 0 |
|  | Less than 1 year | 1.121 | 0.782 | 1.606 | 0.534 |
|  | 1-5 years | 0.950 | 0.593 | 1.519 | 0.829 |
|  | 6-10 years | 0.989 | 0.479 | 2.043 | 0.976 |
|  | More than 10 years | 0.680 | 0.319 | 1.447 | 0.316 |
| Digital health adoption and transformation score | | 75.064 | 26.763 | 210.541 | **<0.001** |

^*^ A *p*-value of less than 0.05 indicates statistical significance.

**Table S8:** Logistic regression analysis results for the prediction of ethical precaution regarding digital health transformation in clinical research (n=990).

| **Predictor** | **Sub-category** | **OR** | **95% CI. for OR** | | ***p*-value^*^** |
| --- | --- | --- | --- | --- | --- |
|  |  |  | **Lower** | **Upper** |  |
| Gender | Male | 0.666 | 0.491 | 0.904 | **0.009** |
|  | Female (REF) | - | - | - | 0 |
| Age (Years) | | 1.008 | 0.987 | 1.030 | 0.447 |
| Profession | Healthcare providers | 0.689 | 0.419 | 1.133 | 0.143 |
|  | Medical researchers or academic | 0.663 | 0.366 | 1.199 | 0.174 |
|  | Others (REF) | - | - | - | 0 |
| Income level | Lower income | 1.242 | 0.724 | 2.132 | 0.431 |
|  | Middle income | 1.062 | 0.646 | 1.746 | 0.812 |
|  | Upper income (REF) | - | - | - | 0 |
| Educational background | Bachelor degree | 0.893 | 0.477 | 1.674 | 0.725 |
|  | Master degree | 0.990 | 0.568 | 1.727 | 0.973 |
|  | PhD (REF) | - | - | - | 0 |
| Professional sector affiliation | Unemployed (REF) | - | - | - | 0 |
|  | Student or Internship | 0.825 | 0.495 | 1.374 | 0.460 |
|  | Hospital or clinic | 0.854 | 0.490 | 1.486 | 0.576 |
|  | Private business | 1.088 | 0.573 | 2.066 | 0.795 |
|  | Academic or research institution | 1.231 | 0.644 | 2.354 | 0.530 |
|  | Entrepreneurial company or industry | 0.739 | 0.297 | 1.840 | 0.516 |
|  | Government agency | 0.760 | 0.360 | 1.605 | 0.471 |
|  | NGO | 0.617 | 0.206 | 1.848 | 0.389 |
| Professional sector affiliation | Not applicable (REF) | - | - | - | 0 |
|  | Public | 1.232 | 0.794 | 1.913 | 0.351 |
|  | Private | 1.059 | 0.663 | 1.691 | 0.812 |
| English proficiency | Fluent (REF) | - | - | - | 0 |
|  | Proficient | 0.999 | 0.708 | 1.411 | 0.997 |
|  | Basic | 0.884 | 0.587 | 1.330 | 0.554 |
| Digital technologies utilization proficiency | Very high | 0.308 | 0.076 | 1.243 | 0.098 |
|  | High | 0.887 | 0.478 | 1.645 | 0.703 |
|  | Neutral | 0.787 | 0.490 | 1.265 | 0.323 |
|  | Low | 1.358 | 0.879 | 2.100 | 0.168 |
|  | Very low (REF) | - | - | - | 0 |
| Received any formal training or education on digital health topics | Yes | 0.425 | 0.295 | 0.612 | **<0.001** |
|  | No (REF) | - | - | - | 0 |
| Years of experience in research | None (REF) | - | - | - | 0 |
|  | Less than 1 year | 1.167 | 0.818 | 1.667 | 0.394 |
|  | 1-5 years | 0.916 | 0.576 | 1.456 | 0.710 |
|  | 6-10 years | 1.055 | 0.513 | 2.169 | 0.885 |
|  | More than 10 years | 0.968 | 0.460 | 2.036 | 0.931 |
| Digital health adoption and transformation score | | 19.456 | 7.224 | 52.404 | **<0.001** |

^*^ A *p*-value of less than 0.05 indicates statistical significance.
